# Unusually prolonged pemetrexed cytotoxicity in a patient with a lung adenocarcinoma: a case report

**DOI:** 10.1186/s13256-017-1436-7

**Published:** 2017-09-16

**Authors:** Linda Sakhri, Julian Pinsolle, Denis Moro-Sibilot, Hélène Pluchart

**Affiliations:** 1 Institut de Cancérologie Daniel Hollard, Groupe Hospitalier Mutualiste, 124 rue d’Alembert, 38000 Grenoble, France; 2 UM Oncologie Thoracique, Clinique de pneumologie, Pôle Thorax et vaisseaux, Centre Hospitalier Universitaire Michallon, BP217, 38043 Grenoble cedex 9, France; 3 UM Pharmacie Clinique, Pôle Pharmacie, Centre Hospitalier Universitaire Michallon, BP217, 38043 Grenoble cedex 9, France

**Keywords:** Lung cancer, Neobladder, Pemetrexed, Toxicity, Neutropenia

## Abstract

**Background:**

We describe a case of pemetrexed toxicities related to reabsorption by an ileal neobladder, which caused prolonged hematotoxicity and nephrotoxicity.

**Case presentation:**

A 59-year-old white man was diagnosed with metastatic wild-type adenocarcinoma of the upper lobe of his right lung. After a first cycle of cisplatin and pemetrexed, he had unusually prolonged aplasia and acute kidney injury.

The prolonged aplasia was caused by pemetrexed reabsorption by the ileal mucosa of the neobladder as pemetrexed was eliminated renally in an active form and is partly lipophilic.

**Conclusions:**

Pemetrexed may be reabsorbed by the ileal mucosa of the neobladder because of its hydrophobic structure and renal excretion in its active form. Acute urinary retention may maintain this phenomenon. Published data excluded a potential role for cisplatin in this toxicity; furthermore, we could not assess pemetrexed concentrations in the blood or urine as these assay techniques are not validated. Thus, care is needed when giving chemotherapy to patients with a neobladder.

## Background

We present a case of pemetrexed toxicities due to reabsorption by an ileal neobladder that occurred in a 59-year-old white man with wild-type metastatic lung adenocarcinoma. He developed unusually extended aplasia and acute kidney injury after a cycle of cisplatin and pemetrexed. We considered that ileal reabsorption of pemetrexed by the neobladder was responsible for the prolonged hematotoxicity. In fact, ileal mucosa absorption is possible because pemetrexed is partly lipophilic and eliminated in an active form.

## Case presentation

We report a case of a 59-year-old white man with a metastatic adenocarcinoma of the lung diagnosed in April 2013. This tumor was *EGFR* (epidermal growth factor receptor), *ALK* (anaplastic lymphoma kinase), *KRAS* (V-Ki ras2 Kirsten rat sarcoma viral oncogene homolog), *ERBB2* (erb-b2 receptor tyrosine kinase 2), and *B-Raf* (V-raf murine sarcoma viral oncogene homolog B1) wild-types. He had a previous history of radical cystectomy, prostatectomy, and vesiculectomy and received a Hautmann neobladder in 2001. The neobladder was constructed from ~ 70 cm of distal ileum, which was anastomosed to his ureters and urethra. He initiated cisplatin and pemetrexed chemotherapy, and a vitamin B12 and folic acid supplementation had been prescribed prior to the initiation of treatment.

After 10 days of chemotherapy he was hospitalized because of fever above 39.9 °C. A blood sample showed neutropenia > 0.3 G/L and thrombocytopenia > 21 G/L. Thus, amoxicillin-clavulanate plus ciprofloxacin treatment was started.

On the 4th day after hospitalization he had acute renal failure, metabolic acidosis, hyponatremia, and hyperchloremia.

An abdominopelvic computed tomography (CT) scan showed major distension of the neobladder and bilateral pyelocaliceal dilatation. Urethral catheterization collected 2 L of a gelatinous liquid.

**Fig. 1:**
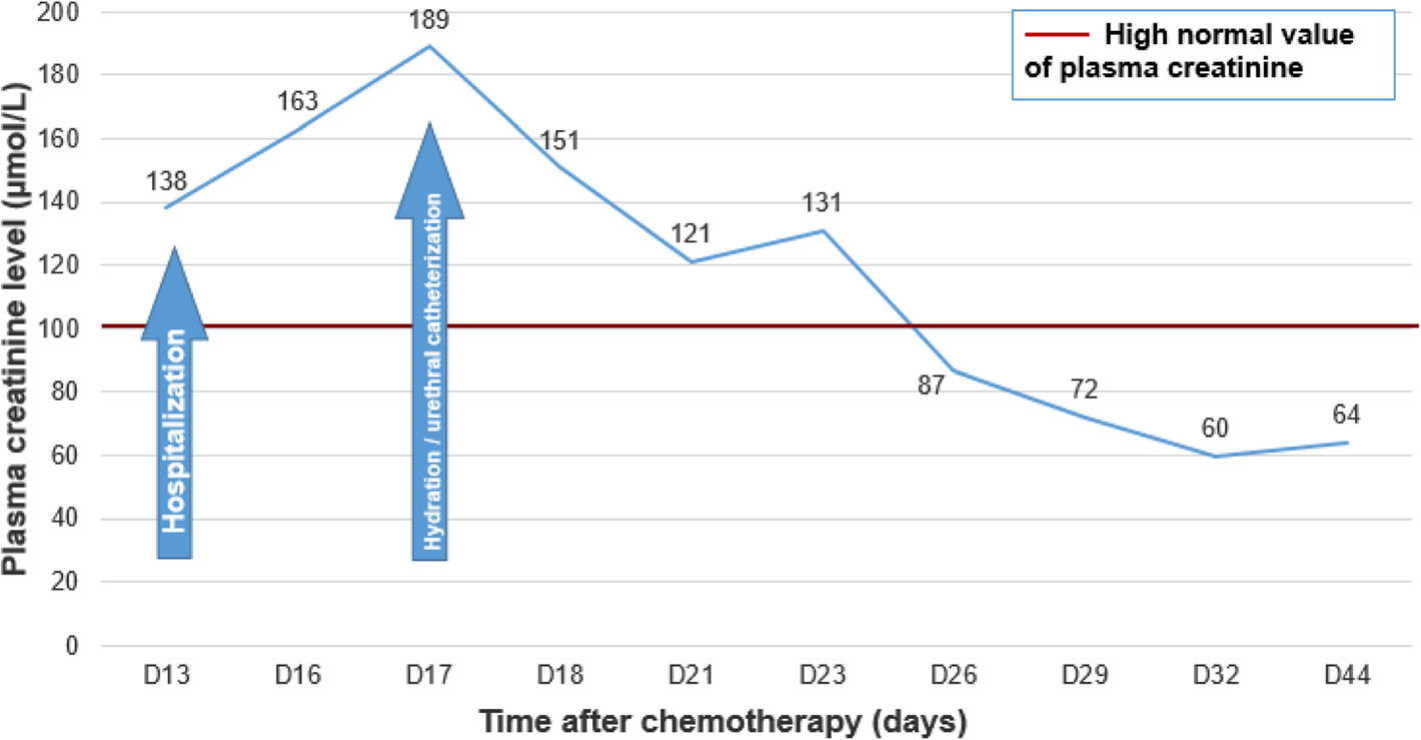
Evolution of plasma creatinine level during the hospitalization

**Fig. 2:**
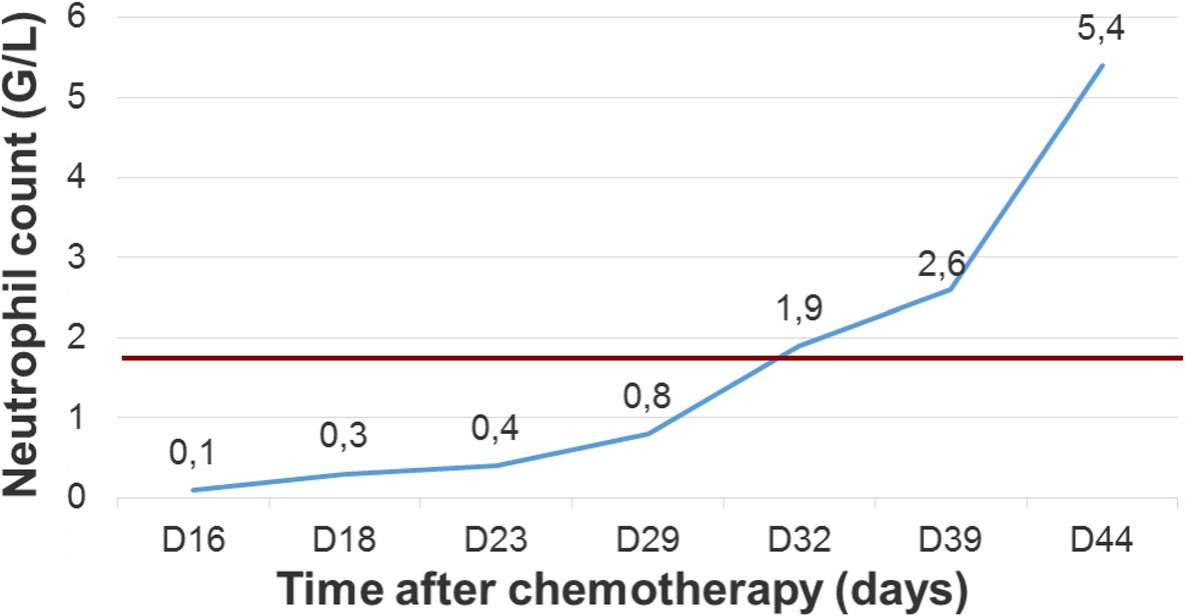
Evolution of neutrophil count during the hospitalization

**Fig. 3:**
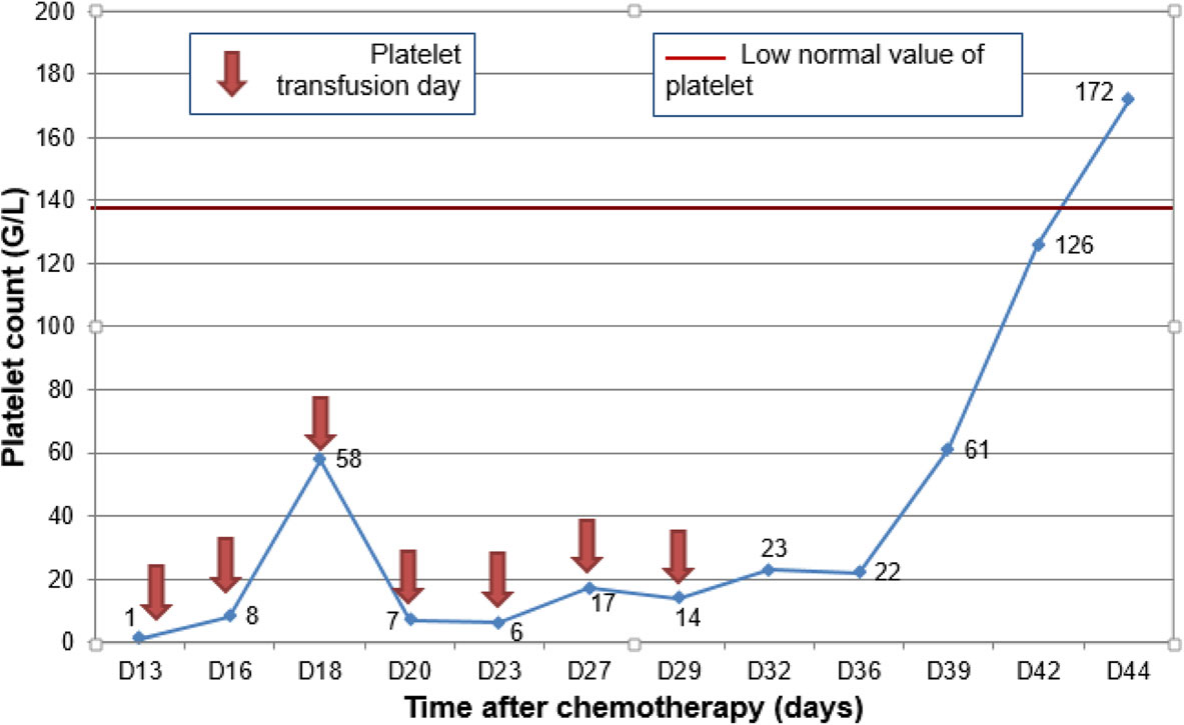
Evolution of platelet count during the hospitalization

The electrolyte disorders were corrected by 23 days after hospitalization and his renal function was restored at 16 days of hospitalization (Fig. 1). His neutrophil levels became standardized by 22 days after hospitalization and platelets by 34 days after hospitalization. He received several transfusions with platelets (Figs. 2 and 3).

## Discussion

Our patient presented unusual hematological adverse effects after a first cycle of chemotherapy. In patients with an advanced stage of non-small-cell lung cancer, pemetrexed is known to cause grade 3 and 4 neutropenia in 15% of cases, grade 3 and 4 thrombocytopenia in 4% of cases [1], and all grades of acute renal failure in 2.4% of cases [2]. In contrast, cisplatin can cause anemia [3], but medullary aplasia is rarely described after only cisplatin [4].

It is to be noted that in patients with locally advanced or metastatic bladder cancer treated with pemetrexed, the hematotoxicity rate is more significant so that cases of grade 3 to 4 neutropenia are up to 75% [5–10]. The hematotoxicity seems more important in patients who had cystectomy. Another study, testing intravesical injections of pemetrexed in pigs, highlighted no myelosuppression and no systemic absorption [11].

Because of the hydrophilicity and the low oral bioavailability of cisplatin, it can be assumed that cisplatin was not absorbed by the ileal mucosa. In a phase I study of pemetrexed, 78% of the administrated dose was found in its active form within the urine. Its plasma half-life is ~ 3.1 hours when renal function is normal [12].

During our patient’s urine-retention episode, we supposed that pemetrexed was stored in an active form by the ileal neobladder due to its lipophilic nature, which maintains toxicities. Cisplatin could not have caused this prolonged toxicity because of its hydrophilicity and so could not be reabsorbed by the ileal mucosa.

## Conclusions

Pemetrexed was the main cause of the adverse effects observed because of its absorption by the ileal neobladder, with this being exacerbated by our patient’s urinary retention.

It was not possible to determine the plasma and urine concentrations of pemetrexed as these techniques are not routinely validated.
